# From genetic to postgenomic determinisms: The role of the environment reconsidered: Introduction to the collection ‘Postgenomic determinisms: Environmental narratives after the century of the gene’

**DOI:** 10.1007/s40656-025-00672-8

**Published:** 2025-04-23

**Authors:** Azita Chellappoo, Jan Baedke, Maurizio Meloni

**Affiliations:** 1 Philosophy Department, The Open University, Milton Keynes, UK; 2 Department of Philosophy I, Ruhr University Bochum, Bochum, Germany; 3 School of Humanities and Social Sciences, Deakin University, Geelong, Australia

**Keywords:** Postgenomics, Determinism, Environment, Epigenetics, Microbiome

## Abstract

In the past twenty years, conceptual and technological shifts in the life sciences have unseated the causal primacy of the gene. The picture emerging from ‘postgenomic’ science is one that emphasises multifactorial dependencies between the environment, development, and the genome, and blurs boundaries between biological individuals, and between the body and the environment. Despite the rejection of genetic determinism within postgenomics, forms of determinism nevertheless persist. The environment is often conceptualised in postgenomic research in a narrow and constrained way, affording an outsized causal role to certain environmental factors while neglecting the influence of others. This carries ethical and social implications, including for understandings of race and motherhood. This topical collection interrogates the environmental determinisms developing within postgenomic science, through investigation of their conceptual foundations, histories, and social contexts across a range of postgenomic fields.

## Introduction

In the last twenty years, significant conceptual and methodological shifts within the life sciences have challenged longstanding perspectives on the concept and status of the gene (Bonduriansky & Day, 2020; Fox Keller, 2000). Technological advances have ushered in what has become known as ‘postgenomic’ science (Richardson & Strevens, 2015). High-throughput whole-genome technologies have enabled the development of fields such as epigenetics, microbiome science, metabolomics, nutrigenomics, and the developmental origins of health and disease framework (DOHaD). Technological developments now allow for cheap and fast sequencing of the genome, the proteome (the collection of proteins expressed by an organism), the transcriptome (the collection of mRNAs), the metabolome (the collection of metabolites at a particular point in time), the hologenome (the collection of genomes of human host and all co-habitating microbiota), and the epigenome (the set of modifications to DNA), illuminating an increasingly fine-grained picture of the complex system of interactions that characterises biological life. Research within environmental epigenetics unseats the gene as the sole bearer of heredity, uncovering pathways through which the environment has a potentially inter- or trans-generational causal influence, through epigenetic modifications to genes. The DOHaD framework attends to the role of these epigenetic mechanisms to link *in utero* or early life exposures to health and disease outcomes across an individual’s lifetime (Pentecost et al., 2024). Moving beyond the human, microbiome science explores the community of microorganisms that inhabit, for example, an organism’s gut or skin, or within soil. As appreciation of the crucial role of the microbiome in physiological and ecological functions grows, boundaries that demarcate biological individuals have become blurred (Chiu & Gilbert, 2015; Gilbert et al., 2012; Skillings, 2016; Raffaetà, 2022).

Within these postgenomic fields, there appears to be a move away from the primacy of the gene, and an accompanying turn against genetic reductionism and genetic determinism (Fox Keller, 2015; Griffiths & Stotz, 2006; Richardson & Stevens, 2015). Postgenomic perspectives refigure development and inheritance as multi-factorial dependencies between environmental factors, developmental mechanisms and the genome (Landecker & Panofsky, 2013; Richardson & Stevens, 2015; Stotz et al., 2006). Thus, the ‘how’ and ‘when’ of genetic information is determined not intrinsically but rather by the genes’ cellular, extra-cellular and extra-organismic environment. In fields like epigenetics, nutrigenomics and microbiome research, the material and social environment– including factors such as stress, toxins, lifestyles, nutritional habits, and income– emerge as shapers of not only humans’ gene expression profiles, but also their ontogenetic and even transgenerational destinies. Here, diseases like cancer and type-2 diabetes, as well as ‘obesity’, autism, and trauma, are conceptualized as instances of social and environmental ‘programming.’ Although this new, more complex, picture appears to hold the capacity to disrupt previous genetic determinist thinking, these developments have simultaneously introduced deterministic or reductionist narratives of their own (Dupras, 2023; Richardson, 2015; Waggoner & Uller, 2015).

Postgenomics has been described as embodying a ‘translational imperative’ (Huss, 2014), referring to the reorganisation of institutions and practices to facilitate a move “more quickly, effectively, and across a wider range of medical problems from bench to clinic” (Maienschein et al., 2008, p. 44). This translational imperative means that the changing landscape of postgenomic science is becoming rapidly deployed in the service of, for example, addressing childhood trauma, developing personalized nutrition or microbiota transplantation technologies, and tackling the purported public health crisis of ‘obesity’.

The unfolding capacity for novel postgenomic science to shape public discourse and policy, and individual lives and behaviour, provides an urgent rationale for interrogating the nature and consequences of emerging postgenomic determinisms. This topical collection explores and interrogates these environmental determinisms from a range of perspectives. Contributions in this collection address the conceptual foundations and theoretical presumptions of different forms of strong causal influences on organisms and human bodies, historically contextualize this new environmentalist trend, and explore the anthropological, social and political contexts affecting and being affected by these developments.

## A short history of environmental determinism

Deterministic narratives about environmental factors in postgenomics– be it social or economic factors, lifestyles and nutritional patterns, toxins, or external influences of other organism like microbes– build on a long, but often forgotten history of similar ideas. These can be roughly distinguished into biological and medical versions of environmental determinism.

In biology, views of environmental determinism can be found arising at least since the mid-19th century, particularly in evolutionary reasoning (Toepfer, 2011). Lamarck and the manifold variants of Lamarckism are the most obvious candidates for a view that gives the environment a leading role in shaping evolutionary traits and sometimes borders on environmental determinism (Meloni, 2016). The position of Darwin is somewhat more complex. On one hand, it is well known that Darwin was highly skeptical of the traditional naturalist view of a direct effect of the environment on the organism’s traits. In fact, with his key notions of indirect effects and random variations, Darwinian selectionism reconfigured the simplistic view of organism and milieu posited by environmentalist models. August Weismann would be probably the most consistent interpreter of this line of enquiry (Meloni, 2017). On the other hand, however, in some (but not in others) of his writing, Darwin suggests that in an environment that does not change, we cannot see changes in organisms’ traits: “If it were possible to expose all the individuals of a species during many generations to absolutely uniform conditions of life, there would be no variability” (Darwin, 1868, II, p. 255). Building on such earlier views, Lloyd Morgan (1892) started using the term ‘environmental determinism’. For Morgan, environmental determinism refers to a mechanism of heredity that involves the transmission of traits individually acquired under the stress of surrounding conditions. He calls this scenario ‘direct environmental determinism’. He distinguished this concept from the inheritance of species-specific characteristics, i.e. ‘innate specific determinism’, and from inheritance based on individual physiological mechanisms, i.e. ‘protoplasmic or physiological determinism.’

In the 1920s environmental determinism began to be more regularly applied to biological contexts, aiming to explain the correlation of organismic characteristics with environmental properties (Bernard, 1924). In his infamously titled book *Apes*,* Men*,* and Morons*, physical anthropologist Hooton (1937) argued that the evolution of all beings except humans is characterized by organismal passivity and environmental determinism. He claimed that in humans we see a significant departure from environmental determinism, emphasizing human evolution as autodirective rather than solely driven by environmental factors. According to Hooton, this shift marked a transition from environmental determinism to self-directed evolution.^1^

From the early 20th century onwards, we see increasing rejections of ideas of environmental determinism in biology. For example, botanist Karl von Goebel (1998/1929) observed that diverse organismal forms thrived under nearly identical environmental conditions. This observation gained significance in the mid-20th century within ecological theories, notably in discussions of the coexistence of competitors (Hutchinson, 1961). Despite this criticism, in the second half of the 20th century we saw trends in evolutionary biology that moved from looking at organism-environment relations as drivers of evolution to unidirectional selective influences on gene frequencies in populations (see Baedke & Fábregas-Tejeda, 2023), sometimes with strong adaptationist claims that characterized organisms as passive and at the mercy of selective pressures (Dawkins, 1976). In this view: “The organism becomes a passive meeting point of forces alien to the very organism” (El-Hani & Emmeche, 2000, p. 235).

In medical contexts the idea of environmental determinism is of course even older and can be seen to date back to some passages of the Hippocratic *On Airs*,* Waters*,* and Places* (5th c BCE) where we can read, for instance, that the inhabitants (of cities that are exposed to the rising of the sun) “have clear voices, and in temper and intellect are superior to those which are exposed to the north, and all the productions of the country in like manner are better” (Hippocrates, 1868, part 5, p. 23). It is important to remember that humoralism combined medical views of the individual body with wider naturalistic understandings of the relationship between environment and human behaviour of whole populations, often with a deterministic undertone exploited for imperial and hierarchical views of human differences. In Roman military treatises for instance (Vegetius, 5th c CE), a deterministic view of the effects of the environmental explained different military qualities among soldiers, with recruits from the North of Europe being seen as more courageous but less intelligent and people ‘from near the sun’ smart but not brave enough to engage in frontal war (a stereotype often applied in the Middle Ages to Oriental populations). In the humoralist tradition, moreover, places are more than embodied: they are inherited by different human groups in ways that are not dissimilar to what we attribute today to genetic factors (see overview of the historical literature in Meloni, 2019; Meloni et al., 2022).

Early modern colonialists largely relied on such views that placed different populations in a geographical hierarchy of values usually linked to the temperate/tropical binary, with the former endowed of the quality of self-control and restraint and the latter associated with lack of civilization, excess and hence incapacity of self-government. The moralistic association of people and places can be seen in the blurred boundaries between notions of *latitude* and *lassitude* (Livingstone, 1991; Bale, 2002). The anxiety of colonizers to embody these degenerative qualities once moving (or being ‘trans- or re-planted’) to tropical places have been largely documented by historians of early modern and modern colonialism (Anderson, 1996, 2006; Earle, 2015). Acclimatization theory is another classic example of a medicine based on the driving force of environmental factors and a paradigmatic science of colonial conquests (Osborne, 2000). It acted as an intellectual bridge between neo- Hippocratic understandings of the body, revived in the 18th century, and later Lamarckian views about the vulnerability of human physiology, and particularly certain races, to different latitudes (Harrison, 1999; Osborne, 2000). Medical astrology, the influence of the moon and sun on fevers and disease can also be cited in this context. While it is usually connected to the work of French or British doctors and naturalists in the 18th century (including Erasmus Darwin, Charles’ grandfather; see Harrison, 2000), the special influence of lunar and solar rays on health and disease has a much older genealogy in Arabic medical astrology since the 8th century (Klein-Franke, 1984). Finally, modern medical geography has also been heavily influenced by a Lamarckian repertoire of imprinting and direct effects of physical places onto human bodies at the individual or group level (Archer, 1993; Campbell & Livingstone, 1983; Peet, 1985).

Other disciplines, including sociology, have long debated the value of environmental forces or ‘milieu’. Although he did not exclude the role of ‘intrinsic nature’, Herbert Spencer brought together biological and social evolution understanding the process of growing complexification as mostly driven by external relations through which organisms (and societies) adapt to the demand of the environing conditions. Life itself was understood by Spencer as “the continuous adjustment of internal relations to external relations”, which were given the driving role (Spencer, 1864, p. 81). Finally, in political theory, since Aristotle’s dichotomy of free Greek and naturally slave Asians (Aristotle, 2013/4th *c* BCE, 1327b) based (also) on climatic factors, environmental determinism has intensely shaped political thinking (Kennedy, 2013). Montesquieu is probably the best-known European example. In his *Spirit of the Laws* (1748/1914, books XIV– XIX) he understood “the empire of the climate” as acting directly on the temperament of people. He connected in this way the power of geography to political institutions establishing a famous binary between hot climates and despotism, on one side, and Northern climates and liberty on the other. This environmental determinism contributed also to justify slavery as a political institution under specific climatic conditions (book XV, ch. 7).

If, as this brief overview has argued here, one of the ongoing source and catalyst for environmental determinism can be traced back to medical humoralism, and considering how medical humoralism influenced the whole of Eurasia for centuries mostly through Persian and Arabic revisitation of the subject, it is not surprising to find four centuries before Montesquieu a similarly complex view on environment and civilizations by Tunis born Arabic scholar Abd al-Rahman Ibn Khaldūn (1332–1406). In his highly influential *Muqaddimah*, Ibn Khaldūn presented a view of the relationship between the environment and civilization based on the existence of seven earth climatic zones, with the coldest and hottest on the two extremes and the mildest in the centre, and the others distributed in between (Gates, 1967; Urban, 2018; Demircioglu, 2014, for a comparison with Montesquieu). The distance from the central mild, Khaldūn assumed, favoured the lack of civilization and actually a whole loss of human qualities, with people in some of the extreme zones were considered similar to animals in their nature. The impact of air on human qualities is also discussed at length in the *Muqaddimah*, giving rise to a racialized typology of human groups based on the relationship between heat, expansion or contraction (in cold) of ‘animal spirits’ and hence higher or lower excitability, capacity to plan future events or just ability to live in the present.

Albeit environmental determinism was not without its critics, and certainly not the only source for racializing thinking in premodern or early modern societies, it has historically shaped for centuries both naturalistic explanations of human differences as well as biological and medical thought. The recent postgenomic move from factors internal to the body to the wider entanglement of bodies and milieux has of course its own specific epistemic and technological features as well as political economy conditions. However, it would not be wrong to consider it as another chapter in a longer history of oscillations between external and internal causes in explaining biological differences among organisms and human groups, and how they change.

## Determinisms from the inside and outside

Throughout the 20th century, this rich history on the biological and medically relevant causal effect of environmental factors has been increasingly overshadowed by a causal emphasis on genes and their roles in development, physiology, evolution and for medical applications. This emphasis has been made in different ways. Among others, genetic determinism follows four main strategies:

i. Narrowing the range of potential causal factors that make a difference for bringing about a certain trait (phenotype).
ii. Overemphasizing the causal strength of single genes (or a set of genes) in causing a trait.
iii. Neglecting other non-genetic (often non-DNA) factors that could make a difference for bringing about a certain trait.
iv. Isolating genetic causal factors from the effects of other cellular, extra-cellular and extra-organismal factors on genes.
v. Highlighting the difference-making capacities of genes or the specificity of genetic effects in contrast to the capacities and effects of non-genetic factors.

Somewhat surprisingly, all of these genetic claims (and combinations of them) can also appear in the form of probabilistic explanations, while remaining nevertheless deterministic in character. This means that a change in a certain gene can be said to deterministically affect (in every organism with that change) the probability to develop a particular trait. For example, in the U.S. approximately 13% of women in the broader population will experience breast cancer at some point in their lifetimes (Howlader et al., 2020). In contrast, 65% of women carrying a BRCA1 variant and 45% of those with a BRCA2 variant are expected to develop breast cancer later in life (Lee et al., 2020). This means that a BRCA1 and BRCA2 mutation determine a particular change in the probability to develop breast cancer. Against this background, Resnik and Vorhaus (2006) distinguish 3 types of genetic determinism: *Strong genetic determinism* asserts that the presence of gene G overwhelmingly results in the manifestation of trait T with a probability of 95% or greater when G is present. *Moderate genetic determinism* suggests that gene G frequently leads to the expression of trait T with a probability exceeding 50% when G is present. *Weak genetic determinism* posits that gene G occasionally leads to the occurrence of trait T with a probability of below 50%.

Strong genetic determinism is rather rare. Doubt against causal models of strong genetic determinism draw on two concepts (see Baedke, 2018, pp. 20–21): First, canalization, i.e. the idea of many-to-one dependencies between genes and their outcomes (Waddington, 1942). For example, when embryos of the fruit fly *Drosophila melanogaster* are cultivated at varying temperatures, the organism’s body segmentation patterns remain constant. Despite this, the expression levels and distribution of the bicoid protein, which typically plays a significant role in body segmentation development, vary (reviewed in Gilbert & Epel, 2009). Canalization underscores the fundamental concept that multiple causes influence a single outcome, thus diminishing the significance of alterations in a single cause. Second, the concept of phenotypic plasticity suggests that an organism’s trait can respond to environmental stimuli in diverse manners (see Minelli & Fusco, 2010; Pigliucci, 2001; West-Eberhard, 2003). This implies that the genome encompasses a broad spectrum of potential phenotypes. For instance, in several dung beetle species, the length of horns and body size in males is influenced by the quality and quantity of food consumed during their larval stage (Moczek, 2010; Moczek & Emlen, 1999). In contrast to oversimplified views of genetic determinism, the concepts of canalization and phenotypic plasticity advocate for a more intricate understanding of the developing organism. This nuanced perspective incorporates multifactorial causation—where many factors influence one outcome—and common causes—where one factor influences many outcomes, or a combination of both: many factors influencing many outcomes.

This more complex view of gene-trait relationships has become standard, or so one may think, in postgenomics. It has been accompanied by a rejection of linear understandings of causality in which organisms are determined internally by their genes. Instead, in recent years, reciprocal causation between organisms and their environment has emerged as a promising conceptual framework (see Baedke & Buklijas, 2023). For example, climate change and Covid-19 vividly demonstrate the profound reciprocal interconnection between our health and the environment. At the same time, in developmental and evolutionary biology, there is renewed emphasis on understanding reciprocal connections between organisms and their environment to comprehend phenomena like developmental plasticity and niche construction. This includes studying organisms’ constructive behaviors as feedback loops that influence natural selection pressures (see Baedke & Fábregas-Tejeda, 2023; Baedke & Gilbert, 2024; Buskell, 2019; Laland et al., 2015; Lewontin, 1982; Odling-Smee et al., 2003). In principle, from these three concepts of canalization, plasticity, and reciprocal causation between organism and environment a view emerges in postgenomics that sees organisms as active and self-constructing agents (see Fábregas-Tejeda et al., 2024; Walsh, 2015). Organisms actively shaping their environments, leading to reciprocal changes in themselves, i.e. changes in gene expression pattern, composition and diversity of their microbiome, changes in biochemical pathways, developmental and behavioural patterns. Instead of merely passively adapting to their local surroundings, organisms dynamically modify their environments to better fit their traits. This shifts the focus from the environment being solely influenced by external factors to being co-constructed by the organism itself.

However, in this more complex postgenomic view, the environment not only takes an important role, but sometime an overly deterministic one. New strategies mirroring those five characteristics of genetic determinism (see above) arise within a postgenomic context. For instance, the narrowing of the range of potential causal factors can also be seen within epigenetics research. One example comes from the maternal-foetal epigenetics research that falls within the DOHaD framework. Richardson (2015) highlights the ways in which DOHaD researchers search for the effects of ‘epigenetic programming’ enacted by the maternal *in utero* environment on the developing foetus. The prenatal period is identified by DOHaD researchers as a crucial period for ‘imprinting’ on the foetus, triggering epigenetic changes that are determinative of later life health outcomes. In this research, the maternal body emerges as an ‘epigenetic vector’, which “converts maternal social conditions into epigenetic marks on the infant’s genome” (Richardson, 2015, p. 219). While there may be a range of features in the environment that causally affect developmental processes, within this range some rather than other social and bodily environments are highlighted, together with specific critical period of development. The maternal body comes to the forefront, constructed as a “narrowly molecularized environment” (van Wichelen & Keaney, 2022, p. 1116; also see Kenney & Müller, 2017; Lappé, 2018; Mansfield & Guthman, 2015; Valdez, 2021; for emerging research on the paternal environment and POHaD paradigm, see Mayes et al., 2022).

As with the neglect of non-genetic factors in the case of genetic determinism, the tendency to neglect certain kinds of factors also features in postgenomic science. For example, which features of the environment are taken to be relevant for epigenetic changes is constrained: in the case of DOHaD research, emphasis is often placed upon factors amenable to individual intervention, such as maternal nutrition (Pentecost & Meloni, 2020; Saldaña-Tejeda, 2018), rather than broader structural factors. Similarly, in the study of the exposome (the total of an individual’s environmental exposures), macro-social or structural environmental exposures have been “relegated to a secondary status” (Giroux, 2024, p. 8). Shostak and Moinester (2015) describe the stratification that arises from the ways the environment is measured in exposomics, and postgenomics more generally, as creating “regimes of perceptibility”, where particular aspects become more visible and others less visible. Furthermore, the emphasis placed on a narrow set of factors can also come hand-in-hand with strong claims about the causal capacities and strength of these factors. In the case of the microbiome, the claims that the microbiome has a crucial and linear causal relationship, with outcomes including ‘obesity’ and mental health states, has been the subject of criticism (Lynch et al., 2019; O’Malley & Skillings, 2018).

## New developments and vistas for postgenomic knowledge: perils and opportunities^2^

Emerging trends in postgenomic determinism is not only potentially epistemically problematic, but also carries important social, political, and ethical implications. At a time of global violence, the intense ecological crisis, and forcibly displaced populations all over the world leading to traumatizing experiences, postgenomic knowledge deriving mostly from epigenetics and microbiome science has been invoked in the context of these unfolding dramatic ecological and humanitarian crises (see Fuzail et al. 2021; Taki & Melo-Martin, 2021; Merrill et al., 2024; Moll et al., 2024). The use of epigenetic clock technologies to assess the exact age of asylum seekers in Europe for instance has caused a heated debate on the limits and risk of such technologies (Abbott, 2018; Ritz-Timme et al., 2018). More broadly, the application of epigenetic and microbiome findings on vulnerable populations exposed to violence, although still limited in its current deployment, has nonetheless raised an important debate on both the potential and limits of such knowledge (Dajani, 2024; Moser et al., 2023; Serpeloni et al., 2019; Sudheer & Banerjee, 2021; see also Tarnas et al., 2023). As Taki and de Melo-Martin write:

[T]he inclusion of epigenetic endpoints in studying the effects of environmental and life conditions on the health of refugees and asylum seekers can help provide quantitative evidence for exposure to adversity and can promote environmental and social justice initiatives, improved healthcare services, and transgenerational equity […]. On the other side, the information gathered could be used to discriminate against refugees and asylum seekers in various ways, including during legal proceedings. (Taki & de Melo-Martin, 2021, p. 2)

Similarly, microbiome scientists, although aware of the risk, suggest using microbiome knowledge to develop holistic approaches to improve the health of refugees (Fuzail et al., 2021).

In such contexts the importance of developing cross-disciplinary collaborations to mitigate risks of reductionism, reification of findings, oversimplified and mechanistic analysis of the effects of trauma, or views of environmental enrichment, are timely and necessary (Chiapperino, 2021; Keaney et al., 2023; Meloni et al., 2022).

If scientists from the Global North and South and bioethicists are debating the impacts and challenges on research in humanitarian settings (Panter-Brick et al., 2020), the attitude of vulnerable or indigenous communities themselves toward the appropriation of epigenetics findings is complex and also evidences the strategic uses but also limitations of biological knowledge for social and racial justice (see Byrne & Keaney, 2024; Warin et al., 2021; and Keaney et al. in this Topical Collection). It is important to highlight also how postgenomic findings percolate more broadly into policy platforms, humanitarian campaigns, and international institutions in ways that are not always obvious or predictable. Notions of plasticity which are highly informed by epigenetic and DOHaD literature for instance have substantially affected the work of economists and the World Bank itself, through a process that has not only expanded DOHaD range of claims (from medical to behavioural) but has also solidified some of its more discussed hypotheses (Moll et al., 2024). Growing studies on ethnic differences in health also increasingly incorporate methylation markers and often operate in a binary and typological fashion that tend to reinscribe race into the epigenome rather than challenge its construct (Meloni et al., 2022).

In the context of epigenetics and microbiome research that examines racial differences, scholars have highlighted the ways in which this research can reinforce narratives of acquired inferiority, characterise minoritized racial groups solely in terms of damage or disease, or stereotype non-Western populations as ‘primitive’ or ‘pure’ (Baedke & Nieves Delgado, 2019; Chellappoo & Baedke, 2023; Meloni et al., 2022; Nieves Delgado & Baedke, 2021). In addition, postgenomic research directs attention towards interventions, often in the form of novel developments in precision medicine, that act upon epigenetic and microbiomic processes in order to remedy health disparities (Mansfield & Guthman, 2015; Saulnier & Dupras, 2017). However, pursuing this avenue also directs attention away from more prosaic causes of racial health disparities: for example, in the United States, the racial disparity between Black and white newborn mortality is reduced by 58% when Black newborns cared for by Black doctors rather than white doctors (Greenwood et al., 2020). Researchers have also explored the implications of the postgenomic focus on the mother as a critical locus, in terms of responsibilizing and blaming practices, as well as the way this interacts with racial and class hierarchies (Pentecost & Meloni, 2020; Richardson, 2021; Saldaña-Tejeda, 2018; Valdez, 2021).

## The workshop and contributions

Based on the general observation that in recent years there is a worrying increase in deterministic views in postgenomics that replace old determining genetic factors through new epigenetic, microbial or socio-cultural ones, this topical collection commenced with a workshop titled “*Postgenomic Determinisms: Environmental Narratives After the Century of the Gene*”, at Ruhr-University Bochum (25th–26th March 2021). At the workshop 20 international scholars from history and philosophy of science, science and technology studies as well as activists gave 10 talks and 6 posters on the topic. The majority of these presentations formed the foundation of the present topical collection. The final 10 contributions address different facets of deterministic views about environmental and non-genetic factors. They tackle conceptual and theoretical issues (Merlin & Giroux; Núñez Casal; Suárez; Müller & Kenney), provide historical contextualization to this recent development (Buklijas & Al-Gailani; Mayes & Meloni) and critically address social, political and ethical underpinnings and consequences of postgenomic determinism (Moormann; Dodson; Chellappoo; Keaney, Byrne, Warin & Kowal).

The contributions can be roughly grouped based on the postgenomic research field they target: epigenetics, microbiome research, exposomics and developmental biology, and nutrition research.

### Epigenetics

In the contribution entitled “*Refusing epigenetics: Indigeneity and the colonial politics of trauma*”, Jaya Keaney, Henrietta Byrne, Megan Warin and Emma Kowal critically address applications of epigenetic knowledge on Indigenous people and the refusal resulting from this. They demonstrate how environmental epigenetics is increasingly utilized to understand the health impacts on communities affected by historical trauma and structural violence. In this context, epigenetics often views traumatic events and prolonged deprivation as biological ‘exposures’ contributing to intergenerational health issues. By drawing on ethnographic fieldwork conducted in Australia, Keaney and colleagues examine Indigenous responses to epigenetics, revealing strategies of ‘refusal’ to engage with it. This includes views of epigenetics as being irrelevant to anti-colonial efforts and as potentially harmful, because it narrows definitions of colonial harm. Against this background, the authors identify an increasing rejection of epigenetics as well as attempts to challenge its perceived benefits for Indigenous health equity.

In “*Normative implications of postgenomic deterministic narratives: The case study of epigenetic harm*”, Emma Moormann provides an analysis of the concept of epigenetic harm. Moormann explores the notion of epigenetic harm as a crucial bridging concept between the relevant scientific findings and growing ethical and legal discussions around epigenetic science, including attributions of epigenetic responsibility. Moormann proposes a multidimensional and flexible understanding of epigenetic harm, that takes into account not only causal pathways, but also lived experience and social and power relations. Nuancing and broadening our understanding of epigenetic harm furthers debates around identifying this harm and ascribing responsibility, and it reduces our vulnerability to deterministic narratives.

Finally, in “*The evolution of ACEs: From coping behaviors to epigenetics as explanatory frameworks for the biology of adverse childhood experiences*”, Ruth Müller and Martha Kenney offer a careful and insightful reading of explanatory frameworks for ‘Adverse childhood experiences (ACEs)’– a trajectory that over time has moved from coping behaviors (1998–2005) to allostatic loads (2005–2013) to, these days, epigenetic factors. The article addresses shifts in the moral and social implications of these different explanatory frameworks particularly for notions of responsibility and intervention. Between notions of molecular evidence and possible reversibility of effects, epigenetic discourses and ACEs explanations are today entering into a relationship of two-way consolidation.

### Microbiome

The paper by Javier Suárez entitled “*Scrutinizing microbiome determinism: Why deterministic hypotheses about the microbiome are conceptually ungrounded*” explores determinism in modern microbiome research. Specifically, he contends that certain views of gene determinism from the Human Genome Project were broadened to investigate causal relationships among symbiotic organisms in microbiome research. Suárez distinguishes between two types of determinism in the field: One perspective suggests that microbiome assembly is chiefly influenced by host genetics, termed ‘host-microbiome determinism’. Conversely, another viewpoint asserts that the microbiome significantly shapes aspects of the host phenotype, termed ‘microbiome-phenotype determinism’, wherein it holds causal precedence over other factors in trait expression. Suárez argues that current evidence does not support these deterministic hypotheses regarding the expression of phenotypic traits in holobionts and the role of the host genotype in assembling the microbiome.

In “*Race and indigeneity in human microbiome science: Microbiomisation and the historiality of otherness*”, Andrea Núñez Casal uses ethnographic fieldwork of a transnational community of microbiome scientists to trace the key actors, experimental systems and methodologies, and underlying ontologies in the foundational beginnings of human microbiome science. Through examination of the ‘Microbiomes of Homes across Cultures’ project and its associated personalised medicine initiative, the ‘American Gut Project’, Núñez Casal reveals the ways in which race is already folded into the research process from its inception. This contribution further develops the concept of the ‘microbiomisation of race’, as constituted by the practices of bioprospection and the ‘remining’ or commodification of bioprospected data.

### Exposomics and development

Francesca Merlin and Élodie Giroux, in “*Narratives in exposomics: A reversed heuristic determinism?*”, offer a philosophical analysis of deterministic narratives in exposomics. They analyse the ways in which causes and effects are characterised and measured within this field and develop a taxonomy of historical conceptions of determinism. They find that narratives emergent in exposomics research can be characterised as deterministic in a Laplacian sense– that is, based on the ideal of completeness. However, this is more applicable to the promises or rhetoric of exposomics than realistic scientific goals. However, methodological determinism plays a crucial heuristic role in exposomics within the research practices themselves.

“*A fetus in the world: Physiology*,* epidemiology*,* and the making of fetal origins of adult disease*” by Tatjana Buklijas and Salim Al-Gailani offers a historical perspective on the field of DOHaD research. DOHaD origins were interdisciplinary, drawing on animal, clinical, and epidemiological tools and attending to the wider social environment. Over time DOHaD’s focus became narrowed, towards the body of the individual mother, which has drawn significant criticism. Buklijas and Al-Gailani argue that this narrowing of focus emerged from the alliance made between David Barker, the epidemiologist who established DOHaD as a field, and fetal physiologists. This connection turned the maternal in utero environment into the focal point of research and marginalised factors such as paternal contributions and structural determinants of health.

### Nutrition

Christopher Mayes and Maurizio Meloni focus in their paper “*Forgetting how we ate: Personalised nutrition and the strategic uses of history*” on the long history of nutritional advices leading to today’s personalized nutrition. They first describe how the fields rejects the one-size-fits-all approach of traditional nutrition, and how their advocates assert that dietary guidance should be tailored to individuals rather than being universally applied. Then, they focus on the historical roots of personalized dietary regimes, starting with Galen and Ibn Sina. They criticize that proponents of personalized nutrition often overlook or misinterpret this history to emphasize the field’s novelty, potentially leading to the commodification of molecular advice and disregarding longer histories of food management in diverse cultures. They seeks to provide a nuanced understanding of this history to challenge the hype and exceptionalism surrounding contemporary personalised nutrition claims.

In “*Postgenomic understandings of fatness and metabolism*”, Azita Chellappoo provides a philosophical analysis of deterministic narratives at play in metabolomics research on ‘obesity’. She outlines deterministic models of ‘obesity’ that have been operative in ‘obesity’ science prior to postgenomics, shaping understandings of ‘obesity’ or fatness: firstly, the model of ‘obesity’ as being determined by the balance of calorie intake to calorie expenditure, and secondly, the model of ‘obesity’ as inherently unhealthy or pathological. Metabolomics carries the promise of disrupting the first model, in that metabolomics tools can elucidate the dynamic and heterogenous nature of human metabolism. However, she argues that the environment becomes fragmented and reduced in metabolomics research, maintaining a disproportionate focus on the metabolic effects of calorie intake and calorie expenditure. Additionally, the model of ‘obesity’ as inherently pathological remains entrenched within metabolomics, reflected in methodological choices and systems of classification.

Finally, the commentary “*Obesogenic vs. fatphobic: An examination of environment in relation to fatness*” provides an important perspective on the real world harms of narratives of environmental determinism. Tiana Dodson, a body liberation facilitator and activist, draws on her lived experience and work within the field of fat studies to explore the ways in which the conception of the ‘obesogenic’ environment as the main causal driver of larger body size continues to pathologise fatness and reinscribe social hierarchies. The narrow conception of the causal pathways between the environment, body size, and health outcomes neglects factors such as stress, discrimination, and stigma, which meaningfully contribute to the quality of life and health outcomes of fat people.

## Conclusions

Genetic determinism has had tremendous influence over the course of the 20th century, and continues to play a role in public understandings of biology, although it is waning within science itself. The shortcomings of deterministic narratives that emphasise the causal primacy of the gene have been thoroughly exposed, including by the technological and conceptual shifts within the life sciences that have emphasised multi-factorial dependencies between the environment, development, and the genome, and reconceived of the body-environment and biological-social boundaries as porous and dynamic (Fox Keller, 2015; Lock, 2012; Meloni, 2018; Richardson & Stevens, 2015; Stotz et al., 2006). This postgenomic shift has dethroned the gene as the primary causal actor, perhaps proving to be the final ‘nail in the coffin’ for genetic determinism (Bjorklund, 2018).

However, determinism in another form persists within postgenomic science. Environmental determinism is emerging in fields such as environmental epigenetics, microbiome science, DOHaD, personalized nutrition, and metabolomics: the material and social environment as conceptualised and operationalised in postgenomic research is often fragmented and reduced (Niewöhner & Lock, 2018). A narrowly conceived environment, such as the maternal uterine environment, can emerge as causally privileged and the predominant focus of research, while other aspects of the environment are backgrounded or neglected (Warin et al., 2011). The abstraction of the environment into data technologies and digital knowledge infrastructures also raises important questions and challenges (Barchetta & Raffaetà, 2024). Finally, the incorporation of developmental plasticity into health models economics, particularly to explain gaps or delays in the growth of human capital in the Global South, brings to light a literature where ‘the environment’ is equated with the accumulated effects of shocks that mechanistically and in a unilinear fashion impinge on allegedly damaged populations (Moll et al., 2024). In sum, while environmental determinism is not new, and has longer histories in both biology and medicine, it is taking on specific and unanticipated forms in 21st century science.

Environmental determinism can have potentially profound social and ethical implications. Consequences include the reinscription of race and narratives of racial inferiority, classed and gendered practices of responsibility attribution and blame, and shifting focus away from addressing broad structural inequalities. Given the role that postgenomic science plays in developing interventions, as well as informing public health policy and wider discourse, it is crucial that its deterministic strands are interrogated and understood. This topical collection aims to contribute to this endeavour, through analysis of the conceptual foundations, histories, and social contexts of postgenomic science across a range of disciplines.

## Footnotes

1 At the same time, in some of his work, Hooton endorsed scientific racism and racial hierarchies, which begs the question if, for him, all humans equally partake in autodirective evolution.

2 we thank Natasha Rooney (Deakin University) for research on this section
